# NSD2 as a Promising Target in Hematological Disorders

**DOI:** 10.3390/ijms231911075

**Published:** 2022-09-21

**Authors:** Alba Azagra, César Cobaleda

**Affiliations:** Immune System Development and Function Unit, Centro de Biología Molecular Severo Ochoa (CSIC–Universidad Autónoma de Madrid), 28049 Madrid, Spain

**Keywords:** epigenetics, leukemia, histone methylation, NSD2, WHSC1, MMSET, SET domain

## Abstract

Alterations of the epigenetic machinery are critically involved in cancer development and maintenance; therefore, the proteins in charge of the generation of epigenetic modifications are being actively studied as potential targets for anticancer therapies. A very important and widespread epigenetic mark is the dimethylation of Histone 3 in Lysine 36 (H3K36me2). Until recently, it was considered as merely an intermediate towards the generation of the trimethylated form, but recent data support a more specific role in many aspects of genome regulation. H3K36 dimethylation is mainly carried out by proteins of the Nuclear SET Domain (NSD) family, among which NSD2 is one of the most relevant members with a key role in normal hematopoietic development. Consequently, NSD2 is frequently altered in several types of tumors—especially in hematological malignancies. Herein, we discuss the role of NSD2 in these pathological processes, and we review the most recent findings in the development of new compounds aimed against the oncogenic forms of this novel anticancer candidate.

## 1. Introduction

From a molecular point of view, the term “epigenetics” refers to the mechanisms that regulate the generation, maintenance, and transmission of gene expression and repression patterns without modifying the DNA sequence; this is achieved by the enzymatic addition of specific chemical residues to the different components of chromatin [1,2,3,4,5,6]. These epigenetic modifications ensure that each gene is expressed (or not) at the right time in the correct cellular context; therefore, since they determine which genes are active at any given time, epigenetic marks are in charge of establishing both cell identity and cell function during development. At the chromatin level, epigenetic control is based on an array of chemical modifications of DNA and histones (methylation, phosphorylation, etc.) that can be either added (“written”) or removed (“erased”) by various epigenetic modifier proteins, and can afterwards be “read” by different effector proteins [7,8]. The combination of these marks gives rise to a code (frequently called the ‘‘histone code’’) that specifies the levels of expression or repression of the different genes at the different cellular stages of development. The most important molecular epigenetic regulators are DNA methylation, chromatin remodelers, non-coding RNAs, and, the focus of this review, histone modifications.

The different types of histones can experience many kinds of post-translational modifications (acetylation, phosphorylation, methylation, ubiquitination, SUMOylation, etc.) affecting different amino acid residues along the histone polypeptide chain (Lys, Ser, Thr, Arg), to different degrees (for example, mono-, di-, or trimethylation). In the opposite way, these marks can also be removed by the reciprocal enzymes (demethylases, deacetylases, etc.), therefore altering the interpretation of the code. Interactions between different epigenetic modifications within the same histone molecule or among different ones, and with other marks (DNA methylation, etc.) determine the final functional outcome regarding DNA accessibility and transcription.

Methylation is one of the most important and best characterized histone modifications. Additionally, it is one of the more versatile; for instance, the methylation of histone H3 at lysines 4, 36, or 79 (H3K4, H3K36, and H3K79) is mainly linked to active gene transcription, whereas the methylation of histone H3 at lysines 9 or 27 (H3K9, H3K27), and histone H4 at lysine 20 (H4K20), is usually associated with gene repression [9,10]. In this way, we can also see that some modifications are antagonistic in their effects, and therefore, they are usually mutually exclusive. Methylation of lysines on histones, and in other proteins, is catalyzed by protein lysine methyltransferases (KMTs), and the methyl group can be removed by protein lysine demethylases (KDMs). In the human genome, over 100 KMTs have been predicted, and mass spectrometry studies have suggested that more than 1000 different proteins can be methylated in lysine residues [11,12,13]. In this review, we will mainly focus on a specific modification, which is the methylation of Lysine 36 of Histone 3 (H3K36), and on the physiological and pathological roles of the main enzyme responsible for its dimethylation (H3K36me2), NSD2 (also known as MMSET—Multiple Myeloma SET domain—or WHSC1 –Wolf–Hirschhorn Syndrome Candidate 1–). The methylation of H3K36 exists in three different statuses: mono- (H3K36me), di- (H3K36me2), and trimethylated (H3K36me3); the second one being the most abundant form. H3K36me3 is closely associated with the gene bodies of active genes [14,15], while the H3K36me2 mark is mostly found in intergenic regions and promoters in different cell types, such as sarcoma or myeloma cells [16,17]. H3K36 dimethylation in vivo is mainly catalyzed by four different, but related, KMTS (NSD1, NSD2, NSD3, and ASH1L) (see below) [18], whereas there is only one enzyme capable of synthesizing H3K36me3 in vivo, and that is SETD2. The protein capable of catalyzing H3K36me1 generation is still unknown, although it is probable that it is generated through the combined actions of KMTs and KDMs. The known H3K36 histone demethylases, which oppose the action of the methyltransferases, belong to the Jumonji C (JmjC) domain-containing family of histone demethylases (JHDMs) [19]. Specifically, there are two JHDMs capable of demethylating H3K36: JHDM1 (also known as KDM2A-B), which acts on H3K36me1 and H3K36me2, and JHDM3 (JMJD2/KDM4A-D), which demethylates H3K36me2 and H3K36me3 [20]. As we will see, H3K36 methylation is involved in several crucial biological processes including chromatin organization [21], transcripts processing and alternative splicing [22,23,24], and DNA damage repair [25,26].

## 2. Deregulation of H3K6methylation and Cancer

The degree of methylation of H3K36 results in different biological consequences, and mutations in the enzymes controlling this mark can result in diverse developmental disorders, and cancer [11]. SETD2 (generating H3K36me3) has been shown to regulate DNA methylation, transcription, RNA processing, RNA epigenetics marks-like N6-Methyladenosine (m6A), and DNA repair [27,28,29,30,31]. How this deregulation of SETD2 gives rise to cancer in many different tissues has already been extensively documented elsewhere [11,32,33,34,35,36,37,38]. In contrast to these outcomes caused by the alterations in the H3K36me3 deposition, the molecular consequences of H3K36 dimethylation are less understood; until recently, it was simply considered a step towards trimethylation, but it is now clear that it has specific roles in itself, and it has been linked to DNA methylation, gene activation, or cellular transformation, for example [16,39,40]. In contrast to the well-established role of SETD2 as a tumor suppressor, the enzymes involved in generating the H3K36me2 mark are primarily active oncogenes upon activating mutations/translocations and, conversely, are mainly associated with developmental problems when completely or partially inactivated [18].

The disruption of epigenetic mechanisms can lead to altered gene function and malignant cellular transformation. In fact, the alterations of the epigenetic landscape constitute a hallmark of cancer [41]. Given the relevant role of the H3K36me2/3 marks in different biological processes, their alteration can be involved in the initiation or maintenance of different types of malignancies [42,43]. For instance, an enrichment of H3K36me3 in oncogenes is tightly related to the aberrant overexpression of these genes in cancer cells [44]. Here, we will focus on the normal and pathological functions of NSD2, which is involved in the initiation and relapse of a wide range of cancers. The altered expression of NSD2 can affect global histone methylation enrichment and high order chromatin organization, leading to the development of diseases such as multiple myeloma or lung cancer [45,46,47]. In myeloma cells, H3K36 methylation is globally increased (in fact, NSD2 was first identified as MMSET—Multiple Myeloma SET domain protein—in human disease [48]), and consequently, its opposed mark, H3K27me3, decreases, leading to the aberrant activation of genes related to cellular adhesion, cell proliferation, and carcinogenesis [47,49,50,51]. In lung cancer patients, NSD2 overexpression and uncontrolled H3K36me2 enrichment lead to rapid tumor progression and also to a poorer outcome, and NSD2 inhibition disrupts the activation of RAS-target genes, contributing to the inhibition of the RAS-mediated oncogenic transcriptional programs [45,52].

Altered H3K36 methylation also impairs the proper differentiation of mesenchymal progenitor cells, leading to sarcomagenesis [53]. NSD2 is also highly expressed in metastatic prostate cancer, where its repression can inhibit metastasis [54], so that NSD2 has been proposed as a key driver of the tumor, and therefore, as a critical therapeutic target for this disease.

## 3. NSD2 Protein: General Features

**Figure 1:**
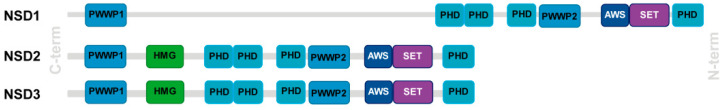
Schematic representation of NSD family proteins basic structure: PWWP, methylation-binding domain; PHD, Plant HomeoDomain; SET, histone methyl transferase domain; AWS, domain associated with the SET domain.

The members of the NSD (Nuclear SET Domain) protein family are structurally conserved methyltransferases [18,40]; the family is formed by NSD1, NSD2, and NSD3 (also known as WHSC1L1—Wolf–Hirschhorn Syndrome Candidate 1-Like 1–) (Figure 1). They present several isoforms that significantly vary in length; the longest isoforms are large proteins containing many defined domains, amongst which the most relevant one is the methyltransferase catalytic domain SET (Su(var)3-9, Enhancer-of-zeste, Trithorax), which can be further subdivided into pre-SET, SET, and post-SET domains [55,56]. The SET domain catalyzes the transfer of the methyl group from S-adenosylmethionine (SAM) to the substrate protein, and propagates the H3K36me2 active mark to nearby nucleosomes; apparently, it is also capable of trimethylating H3K36 in the presence of SETDB1 [57,58]. Additionally, full-length isoforms have, in the amino-terminal portion, a high-mobility-group (HMG) box, two PWWP domains necessary for binding to methylated Histone 3, as well as the DNA and PHD (plant-homeodomain) domains necessary for the interactions with other modified histones [59,60,61] (Figure 1). These multiple protein–protein interaction (PPI) modules can act as potential chromatin readers, and the evidence indicates that they play important roles in NSD protein function, although their precise functions are still being elucidated [62]. PWWP domains are known to possess the capacity of reading the presence of H3K36-me2 or -me3 marks while, at the same time, binding the nucleosomal DNA besides the H3K36 residue [63,64]. The most N-terminal domain of NSD2 can bind to nucleosomes di- and tri-methylated in H3K36, supposedly stabilizing NSD2 binding to chromatin [61]. 

NSD1 and NSD2 are crucial for proper murine embryonic development, since their knockouts are embryonic lethal [65,66], and NSD3 is critical for correct neural crest development [67,68]. In humans, congenital heterozygous mutations of NSD1 cause Sotos syndrome, a severe developmental defect occurring in 1/14,000 births and causing excessive growth, advanced bone age, a characteristic face, neurological disorders, brain abnormalities, and intellectual disability [69]. Similarly, a hemizygous chromosomal deletion affecting NSD2 causes Wolf–Hirschhorn syndrome (WHS), characterized by growth deficiency, immunodeficiencies, a characteristic facial appearance, intellectual disability, and seizures [70,71,72].

NSD proteins can methylate in vitro many different histone residues: H3K4, H3K9 H3K27, H3K36, H3K79, and H4K20; however, in vivo, their function is largely restricted to the mono- and di-methylation of H3K36, and to a much lesser degree, H4K20 [47,73,74]. One important structural particularity of the NSD proteins, which present a major challenge for the development of specific inhibitors [75], is the presence of an autoinhibitory loop, which connects the SET and post-SET domains and blocks the catalytic domain in the substrate-unbound enzyme [76,77], in such a way that it is only accessible after a conformational change triggered by the binding to the nucleosomal DNA [11,73]. Therefore, the development of inhibitors will very likely require screening designs performed in the presence of assembled nucleosomes.

## 4. NSD2 Mutations and Their Oncogenic Consequences

NSD2 is one of the most frequently mutated epigenetic regulators in several cancer types, especially pediatric cancers [78]. Different types of mutations have been described in NSD2, which are associated with several human pathologies. As mentioned, an insufficiency of NSD2 is established as one of the main causes of Wolf–Hirschhorn syndrome, and its associated defects in B cell development and immune function [70,71,72,79]. On the contrary, NSD2 hyper-activation caused by either point mutations or genomic translocations represents a relevant cause of B-cell-associated hematological malignancies, such as multiple myeloma and acute leukemia, and other types of cancers including colon cancer and lung carcinoma [45,78,80,81,82].

**Figure 2:**
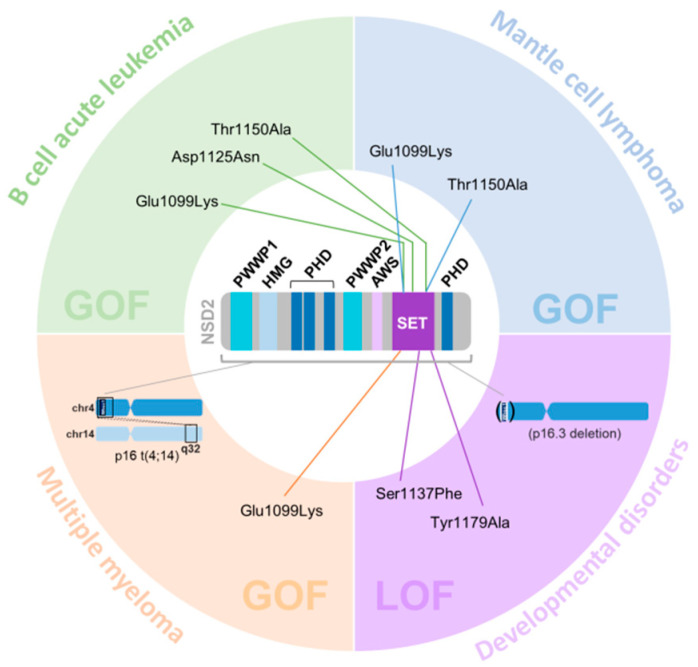
Genetic alterations of *NSD2* associated with hematological diseases. Point mutations at the SET catalytic domain and chromosome aberrations are indicated. Hematological malignancies (B cell acute leukemia, mantle B cell lymphoma, and multiple myeloma) are mainly associated with NSD2 gain of function (GOF) alterations, while developmental disorders (Rauch–Steindl syndrome and Wolf–Hirschhorn syndrome) are associated with NSD2 loss of function (LOF) alterations.

Focusing on the NSD2 mutations found in hematopoietic pathologies and malignancies (Figure 2), these could be classified into:

### 4.1. Gain-of-Function Mutations

#### 4.1.1. Acute Lymphoblastic Leukemia

B-cell acute lymphoblastic leukemia (B-ALL) is a clonal malignant disease that originates in a single cell and is characterized by an accumulation of immature B-cells that are phenotypically reminiscent of the normal stages of B-cell differentiation. This alteration finally leads to the suppression of normal hematopoiesis and the infiltration of many vital organs [83]. Relapsed B-cell Acute Lymphoblastic Leukemia (B-ALL) remains a leading cause of cancer mortality in children [83,84], accounting for 15–20% of the pediatric cases. Intriguingly, the mutations of epigenetic modifiers are found in a great amount of patients at relapse, suggesting a role for the epigenome in disease progression and therapy resistance [78,84,85,86]. NSD2 stands out as one of the genes mutated in a relapse-specific manner, with different mutations in the catalytic SET domain, among which p.Glu1099Lys (E1099K) is the most recurrent, leading to leukemic cell proliferation [80]. NSD2 mutated subclones at early stages of the disease results into “first settler” clones at relapse [86]. This NSD2^E1099K^ mutation is enriched in a B-cell leukemia subgroup characterized by the presence of the fusion gene *ETV6-RUNX1* as a primary oncogenic lesion [80].

Point mutations taking place in E1099K, D1125N, and T1150A NSD2 positions enhance the interaction with chromatin nucleosomes, producing enzyme hyperactivity [80,87,88,89,90,91,92,93,94,95,96,97,98,99]. Specifically, E1099K and T1150A mutations destabilize the auto-inhibitory loop located in the catalytic site of the enzyme, keeping it in an open state and therefore promoting the enhancement of its catalytic activity [88]. Compared to control cells, those cells carrying the E1099K mutation present increased H3K36me2 enrichment, with a parallel decrease in the antagonistic mark H3K27me3; this causes a 3D genome reorganization that maintains the open chromatin state compartments and leads to higher proliferation rates [87,90]. Thus, alterations in this epigenetic regulator trigger epigenetic changes, higher order chromatin reorganization, changes in genes expression, and finally, phenotypical consequences such as aberrant cell proliferation capacity.

All these molecular mechanisms by which NSD2 confers treatment resistance represent an essential stage for disease relapse and are still under study. The global chromatin reorganization suffered by ALL cells due to NSD2 hyper-activation points towards epigenetic landscape modification as one of the main regulators of cellular fitness and response to the environment [84]. More recent studies using high-throughput drug screenings have unveiled that uncontrolled NSD2 activity caused by E1099K mutation is related to glucocorticoids resistance [91]. The uncontrolled cell growth and clone enrichment at relapse reinforce the potential role of NDS2 as an oncogene, and as a potential target for the development of specific inhibitors for efficient leukemia treatment. 

#### 4.1.2. Multiple Myeloma

Multiple myeloma (MM) is a hematological malignancy characterized by the abnormal accumulation of clonal plasma cells in the bone marrow [92]. MM accounts for 10% of hematological malignancies and 1.6% of all U.S. cancer deaths. The median age at diagnosis is 69 years. Genetic aberrations are observed from the early stages of the disease and are key events in the establishment of the clonal plasma cell population. MM can be classified into two major subtypes: hyperdiploid and non-hyperdiploid. The second type is mainly characterized by translocations leading to the activation of proto-oncogenes located in multiple partner chromosomes. Approximately 15–20% of MM patients carry a translocation in the chromosome 4 (t(4;14)) that puts NSD2 expression under the control of the immunoglobulin heavy chain locus, leading to its overexpression, and a consequent global increase in H3K36me2, coupled with a global H3K27me3 decrease through EZH2 inhibition [16,17,48]. In fact, the disorganization in H3K36me2 distribution is one of the first steps of the myeloma oncogenic program, due to the alteration in gene expression patterns involved in cell growth, adhesion, chromatin accessibility, and DNA damage response [16,47,49,51,93]. Additionally, the DNA damage repair capacity of cells overexpressing NSD2 could explain their resistance to treatment [94]. C-MYC stands out among the genes with altered expression levels upon NSD2 overexpression, attributed to miR126 aberrant repression [95]. In fact, TP53 and C-MYC have been postulated as key targets of NSD2 in MM, and their altered expressions are directly related with aggressiveness and a poorer outcome in different subsets of myeloma patients (including both NSD2-related myeloma patients and NSD2 non-related ones) [96,97].

However, NSD2 enzymatic activity is not only altered upon chromosome translocation in myeloma patients. There is increasing evidence of myeloma patients that harbor the aforementioned E1099K point mutation, which is not exclusive to ALL patients; also in MM, this mutation leads to NSD2 hyper activation with H3K36me2 global enhancement, H2k27me3 global decrease, and a consequent alteration of the gene expression patterns [81]. 

Given that NSD2 is known to play a relevant role in multiple myeloma relapse and treatment resistance [94,98], genetic and epigenetic compounds should be found capable of inhibiting NSD2 activity regardless of the nature of its alteration. 

#### 4.1.3. Mantle Cell Lymphoma

Mantle cell lymphoma (MCL) is an uncommon incurable and aggressive B-cell lymphoma; it is a heterogeneous disease with variable presentations, and nowadays, it is in fact considered a mixed bag of several subtypes of non-Hodgkin’s lymphomas with a spectrum of molecular and clinical features. NSD2 is one of the most commonly mutated genes in mantle cell lymphoma, and it is associated with poor prognosis [99,100,101]. Interestingly, both E1099K and T1150A mutations are also found in mantle cell lymphoma, associating the disease with the hyperactivity of the enzyme. In fact, the alterations of NSD2 function can be triggered from either E1099K mutation, the T1150A mutation or a combination of both, again leading to the overexpression of genes related to proliferation and cell cycle regulation [87,102]. The T1150A mutation produces the formation of additional hydrogen bonds, which facilitate the insertion of the histone into the catalytic pocket of the enzyme [103]. 

#### 4.1.4. Acute Myeloid Leukemia (AML)

Several cases of human acute leukemia, including both AML and ALL, were linked to SETD2 mutation events, normally associated with additional chromosomal abnormalities [34]. Furthermore, recent studies postulated that AML patients carrying mutations in SETD2 are more resistant to treatment due to the defects in cell cycle checkpoints [104]. Given that SETD2 and NSD2 present a similar catalytic domain, it would be reasonable to hypothesize that mutations in NSD2 could be associated with AML development.

No evidence has been published to date, and preliminary results do not assign poorer outcomes to AML patients from different cohorts stratified according to NSD1 and NSD2 expression levels [105]. However, alterations in NSD1 and NSD2 function have been linked to erythroleukemia development, a subtype of acute myeloid leukemia; indeed, it has been reported that NSD1 is a critical regulator of erythroid differentiation, since its knockdown impaired proper erythroblast maturation and induced erythroleukemia in mice [106]. NSD2 has also been described as a master regulator of erythroid differentiation [107]. In this case, NSD2 expression is inversely correlated to BRCA1 activity and erythroid differentiation in leukemia cell lines. The degradation and loss of NSD2 stability promoted by BRCA1, induces hemin-dependent leukemia cell differentiation [107]. Thus, the interaction between NSD2 and BRCA1 could become a target mechanism to establish new therapies for erythroleukemia. 

#### 4.1.5. Other Hematopoietic Malignancies

In general, the main hematopoietic tumors in which *NSD2* mutations seem to play a significant role in cancer development or evolution are those described in the previous sections. In fact, in a screening of 181 “Cancer Cell Line Encyclopedia” cell lines of hematopoietic origin including chronic myeloid leukemia (), Burkitt’s lymphoma (BL), diffuse large B-cell lymphoma (DLBCL), and Hodgkin’s lymphoma HL (HL) samples, among other hematopoietic cancer types, only six B-ALL cell lines and one myeloma cell line were found to harbor the E1099K alteration, plus eight myeloma cell lines that contained the t(4;14) translocation [80]. A larger screening of patient’s samples, part of the American Association for Cancer Research “GENIE” initiative [108], has recently identified occasional mutations of *NSD2* in small percentages of hematologic cancers such as non-Hodgkin lymphomas (*NSD2* is altered in 2.86% of the patients), Hodgkin lymphoma (1.79%), diffuse large B-cell lymphoma (2.58%), or chronic lymphocytic leukemia/small lymphocytic lymphoma (0.53%) [108]. These small percentages are in reasonable agreement with those found in the COSMIC database [109] and seem to suggest that, although *NSD2* mutations can be involved in the tumor progression in many different types of hematopoietic malignancies, its most relevant pathological role is played in the context of B-ALL, MM, and MCL.

### 4.2. Loss-of-Function Mutations

As previously mentioned, the hemizygous loss of NSD2 is related par excellence to the Wolf–Hirschhorn Syndrome (WHS), where a variable segment of chromosome 4 is lost, including the *WHSC1*-containing region [65,70]; WHS is a rare disease, with a prevalence of 1:20,000/50,000 worldwide, which affects the whole organism causing severe developmental defects, including failures in antibody production and other lymphocyte functions [110]. 

Patients with developmental disorders other than WHS can also carry NSD2 loss-of-function mutations, such as S1137F or Y1179A, which impair the enzyme catalytic activity and consequently, diminish H3K36 dimethylation levels [111,112]. The fact that the symptoms from patients carrying these mutations do not completely overlap with those of WHS patients suggests that the molecular consequences of H3K36me2 loss caused either by NSD2 deletion or by loss-of-function mutations present some differences that will need further research [111,112]. 

Regarding the implications of NSD2 loss-of-function in cancer, it has been reported that it can also have tumor suppressor functions controlling progenitor cell differentiation. It has been reported that, upon NSD2 knockout, zebrafish presented developmental defects resembling Wolf–Hirschhorn Syndrome; however, at later stages, these animals developed swim bladder tumors, indicating a potential tumor suppressor function of NSD2 [113].

## 5. NSD2 Inhibitors and Therapeutic Strategies

Many histone lysine methyl-transferases have been highlighted as promising therapeutic targets in cancer, and members of the NSD family are not an exception. As previously explained, their activating mutations and up-regulation seem to drive both tumor progression and treatment resistance in several types of cancer. Accordingly, extensive research has been conducted in recent years in order to identify the different selective inhibitors to target the catalytic domain of NSD proteins [87] (Table 1).

Given that NDS proteins contain a catalytic SET domain shared with other proteins included in the same super family of histone methyltransferases, some NSD inhibitors were initially reported as inhibitors for other SET domain-containing enzymes; for example, BIX-01294 was initially identified as a G9a-like protein (GLP) inhibitor, capable of reducing H3K9me2 levels during induced cell reprogramming [114,115]. BIX-01294 binds to the enzymatic SET domain of GLP and similarly, to the SET domain of NSD, obtaining in fact a better inhibition rate on NSD2 protein [116]. This finding contributed to the study of NSD proteins biology and to the development of selective NSD inhibitors, but more extensive research is required to increase target specificity. 

Sinefungin, a natural nucleoside isolated from *Streptomyces* and related to S-adenosylmethionine, has been widely postulated as a nonselective inhibitor of the SET domain activity; it was first tested on SETD2 [117], and then the potential of sinefungin analogues for NSD2 inhibition was also tested. Despite the fact that these proteins present slight differences in their substrate binding pockets and their SET domains’ 3D organization, the findings provided a helpful contribution for further research on the selective inhibitors of NSD2 protein [118]. Along the same lines, given the similarity in function and structural conservation among the different H3K36me2 methyltransferases, it would be reasonable to apply the therapeutic agents designed against one enzyme to the others, thereby reducing the development of new inhibitors for the same kind of targets. For example, one could use the recently developed ASHL1 inhibitors in cases of NSD2 hyperactivation [119]. However, even though NSD2 and ASHL1 both present the autoinhibitory loop in the SET domain, which would be necessary for this type of inhibition, the sequence of this loop is poorly conserved among proteins and restricts the inhibition effectivity to only AHSL1 [119,120].

The first validated peptide specific inhibitor against the catalytic activity of NSD2 in multiple myeloma cases was PTD2 [121], whose inhibitor effect is specific in the presence of the cofactor S-adenosyl-L-methionine (SAM). After multiple assays, a useful pipeline for the discovery of selective NSD2 inhibitors by the high-throughput screening of small molecules was developed, which led to the description of five potential inhibitors for NSD2 activity: DA-3003-1, PF-03882845, chaetocin, TC LPA5 4, and ABT-199. All compounds exhibited similar effectivity on either WT or mutated (E1099K and T1150A) NSD2 enzyme forms [122].

Recently, the, Di Luccio laboratory has discovered LEM-06 and LEM-14, which seem to be NSD2-specific inhibitors (with weak activity on NSD1 and NSD3 proteins) [123,124]. They put forward LEM-14 and LEM-14-1189 as promising tools for investigating the biology of the NSDs, and they represent a relevant step in the further development of specific NSD inhibitors used to treat malignancies, including multiple myeloma [124].

Using high-throughput compound screenings, 5-Aminonaphthalene derivatives have emerged very recently as selective inhibitors of NSD2 activity in multiple myeloma and acute lymphoid leukemia cell lines. Cells exhibit increased apoptosis and cell death rates upon the action of these inhibitors, among which compound 9c stands out, which inhibits NSD2 catalytic activity, and consequently, so does the transcriptional activity of NSD2 target genes [125]. 

Since the structure of NSD proteins contains many domains (in addition to the catalytic SET domain itself) that play an important role in protein activity, other approaches are being developed to target methyltransferase activity by interfering with those domains. It has recently been found that the PWWP domains can be themselves druggable, and a chemical probe targeting NSD3 N-terminal PWWP domain can reduce the proliferation of leukemic cell lines [126]. Additionally, an antagonist of the equivalent PWWP domain in NSD2 can abrogate the binding to H3K36me2 [127], therefore proving that targeting other domains beyond the SET could lead to the efficient modulation of NSD2 binding to chromatin and, therefore, its subcellular localization, and perhaps even its catalytic function [62]. Recently, the use of virtual and target class screenings has allowed the identification of a first-in-class chemical probe, UNC6934, that selectively binds an aromatic cage in NSD2-PWWP1, leading to the disruption of its interaction with H3K36me2 nucleosomes [62,127]. UNC6934 can bind full-length NSD2 in a specific and potent manner, and can trigger its dissociation from chromatin, therefore disrupting a cooperative chromatin binding and the methylation mechanism, which seems to require the concerted action of multiple protein domains. 

Finally, more indirect approaches are also possible; for example, inhibitors of the epigenetic repressor complex PRC2 were postulated as promising therapeutic agents against relapsed NSD2-mutated B-ALL cells [91]. The alteration of global histone methylation pattern caused by NSD2 hyperactivation affected the expression of the glucocorticoid receptor (GR), therefore inhibiting the cellular response to glucocorticoid (GC) treatment. Upon PRC2 inhibitor treatment, there was an increase in the number of GRs, hence restoring the response to GC treatment, and activating the expression of pro-apoptotic genes in B-ALL cells.

## 6. Conclusions and Future Perspectives

Somatic mutations were identified in the majority of genes coding for histone methyl transferases in many types of cancers, most of which are resistant to treatment, therefore reinforcing the idea of the influence of the epigenetic and global chromatin landscape in tumor development and relapse.

This review highlights the role of the epigenetic regulator NSD2 in different hematological malignancies. NSD2 was established as a promising target for the treatment of several types of hematological cancers, since both enhanced expression and increased activity caused by either chromosome translocations or different point mutations are involved in tumor progression and treatment resistance. Mutations and deletions disrupting NSD2 function were also linked to disease, but most of the patients present a clinical profile related to developmental disorders, rather than malignant tumors.

In recent years, great efforts have been made to identify the different mutations that affect NSD2 catalytic activity, as well as to develop the selective inhibitors of such activity. Valuable contributions to the discovery and optimization of potent and specific inhibitors of NSD2 have been made in hematological malignancies, especially in acute leukemia, which have greatly contributed to understanding the role of these enzymes in both normal and pathologic development in different types of hematopoietic cells. The most recent research publications are shedding new light on the NSD2-related mechanisms involved in relapse and resistance to treatment, such as the loss of response to glucocorticoids [92]. These findings represent an essential step in the field, but further basic and clinical studies are needed to fully understand the regulation of the H3K36 mark in development and disease, and to refine therapeutic strategies for relapsed cancers.

## Figures and Tables

**Table 1:** NSD2 inhibitors and therapeutic strategies.

Compound	Chemical Name	Specific Target	Therapeutic Aplication	References
BIX-01294	(1H-1,4-diazepin-1-yl)-quinazolin-4-yl amine derivative	GLP and NSD SET domain	Induces cell autophagy (antitumor activity).	[114,115,116]
Sinefungin	delta-(5’-adenosyl) derivative of ornithine (natural compound)	SETD2 and NSD2	Antiparasitic agent. Potential antitumor activity.	[118]
PTD2	(Norleucine-containing peptide)	NSD2	Multiple myeloma treatment	[121]
Chaetocin	14-(hydroxymethyl)-3-[14-(hydroxymethyl)-18-methyl-13,17-dioxo-15,16-dithia-10,12,18-triazapentacyclo[12.2.2.01,12.03,11.04,9]octadeca-4,6,8-trien-3-yl]-18-methyl-15,16-dithia-10,12,18-triazapentacyclo[12.2.2.01,12.03,11.04,9]octadeca-4,6,8-triene-13,17-dione Piperazine (fungal myotoxin)	NSD2 (mutated and WT forms), G9a and SU(VAR)3-9)	Potential antitumor activity	[122]
LEM-06 LEM-14	N-Cyclopropyl-3-oxo-N-(4-pyrimidin-4-ylcarbamoyl)benzyl)-3,4-dihydro-2H-benzo[b][1,4]oxazine-7-carboxamide	NSD2 (NSD1/3 weak)	Antitumor activity (specially multiple myeloma)	[124]
9c	Amino-N-benzylnaphthalene-1-sulfonamide hydrochloride	NSD2 catalytic domain	Multiple myeloma and ALL	[125]
UNC6934	N-Cyclopropyl-3-oxo-N-(4-(pyrimidin-4-ylcarbamoyl)benzyl)-3,4-dihydro-2H-benzo[b][1,4]oxazine-7-carboxamide	NSD2 PWWP1 domain	Potential antitumor activity. Disrupts chromatin–protein interaction	[127]
